# Changing Trends in the Prevalence of *Shigella* Species: Emergence of Multi-Drug Resistant *Shigella sonnei* Biotype g in Bangladesh

**DOI:** 10.1371/journal.pone.0082601

**Published:** 2013-12-18

**Authors:** Abu I. M. S. Ud-Din, Syeda U. H. Wahid, Hasan A. Latif, Mohammad Shahnaij, Mahmuda Akter, Ishrat J. Azmi, Trisheeta N. Hasan, Dilruba Ahmed, Mohammad A. Hossain, Abu S. G. Faruque, Shah M. Faruque, Kaisar A. Talukder

**Affiliations:** Centre for Food and Water Borne Diseases, International Centre for Diarrhoeal Disease Research, Bangladesh, Dhaka, Bangladesh; Institut National de la Recherche Agronomique, France

## Abstract

Shigellosis, caused by *Shigella* species, is a major public health problem in Bangladesh. To determine the prevalence and distribution of different *Shigella* species, we analyzed 10,827 *Shigella* isolates from patients between 2001 and 2011. *S. flexneri* was the predominant species isolated throughout the period. However, the prevalence of *S. flexneri* decreased from 65.7% in 2001 to 47% in 2011, whereas the prevalence of *S. sonnei* increased from 7.2% in 2001 to 25% in 2011. *S. boydii* and *S. dysenteriae* accounted for 17.3% and 7.7% of the isolates respectively throughout the period. Of 200 randomly selected *S. sonnei* isolates for extensive characterization, biotype g strains were predominant (95%) followed by biotype a (5%). Resistance to commonly used antibiotics including trimethoprim-sulfamethoxazole, nalidixic acid, ciprofloxacin, mecillinam and ampicillin was 89.5%, 86.5%, 17%, 10.5%, and 9.5%, respectively. All isolates were susceptible to ceftriaxone, cefotaxime, ceftazidime and imipenem. Ninety-eight percent of the strains had integrons belonging to class 1, 2 or both. The class 1 integron contained only *dfr*A5 gene, whereas among class 2 integron, 16% contained *dhfr*AI-*sat*1-*aad*A1-*orf*X gene cassettes and 84% harbored *dhfr*A1-*sat*2 gene cassettes. Plasmids of ∼5, ∼1.8 and ∼1.4 MDa in size were found in 92% of the strains, whereas only 33% of the strains carried the 120 MDa plasmid. PFGE analysis showed that strains having different integron patterns belonged to different clusters. These results show a changing trend in the prevalence of *Shigella* species with the emergence of multidrug resistant *S. sonnei*. Although *S. flexneri* continues to be the predominant species albeit with reduced prevalence, *S. sonnei* has emerged as the second most prevalent species replacing the earlier dominance by *S. boydii* and *S. dysenteriae* in Bangladesh.

## Introduction

Bacillary dysentery such as shigellosis is endemic throughout the world, and is one of the major causes of morbidity and mortality, especially among children <5 years of age in many developing countries including Bangladesh [1], [2]. Shigellosis is caused by any one of the four species of *Shigella*: *S. dysenteriae, S. flexneri, S. boydii*, and *S. sonnei* and outbreaks caused by *Shigella* infection are difficult to control due to their low infectious dose [3], [4]. Globally, every year there are about 165 million cases of *Shigella* infection and 1.1 million *Shigella*-related deaths. The majority of these cases occur in developing countries [5]. *S. sonnei* is the predominant *Shigella* spp. in developed as well as industrialized countries and is often the second most prevalent *Shigella* spp. in low income countries [5], [6]. In Bangladesh, shigellosis is endemic. Previously, it accounted for 11% of deaths [7], although a recent report suggests that the mortality rate due to shigellosis has decreased to ∼0.01% overall and ∼0.89% among youngest age group [2]. Previously, *S. flexneri* was the predominant serogroup (55%) followed by *S. dysenteriae* (19%), *S. boydii* (13%) and *S. sonnei* (7%) [8].

In developing countries, increased resistance to commonly used antibiotics including ampicillin, streptomycin, sulfamethoxazole-trimethoprim, nalidixic acid and tetracycline has been a major concern in the treatment of enteric infections due to various bacterial pathogens [9]. *Shigella* is transmitted efficiently in low-dose via fecal-oral route in areas of poor hygienic conditions with limited access to clean and potable water [10]. Infections can result from as few as 10 *Shigella* bacteria [3]. Emergence of multidrug-resistant (MDR) strains is increasing rapidly due to their ability to acquire and disseminate exogenous genes associated with mobile genetic elements such as R-plasmids, transposons, integrons, and genomic islands on the bacterial chromosome [11], [12]. Currently, based on the characteristics of integrase genes, five classes of integrons (classes 1, 2, 3, 4, and 5) have been identified [13]. Several reports indicate that integrons have a role in the dissemination of resistance among gram-negative pathogens and are thus a useful marker of antibiotic resistance [14]. Of the five classes, only class 1 and 2 integrons have been found in *Shigella* spp. [12], [15], [16]. With the increased movement of people across national and international borders, there is an increased risk of spread of MDR *Shigella* strains. Following on a recent trend of increased prevalence of *S. sonnei* in Bangladesh (K. A. Talukder *et al*., unpublished data), this study was designed to determine the distribution of *Shigella* species and characterize randomly selected *S. sonnei* isolated between 2001 and 2011 in Bangladesh.

## Materials and Methods

### Bacterial Strains

A total of 10,827 *Shigella* strains were isolated and identified according to standard microbiological and biochemical methods [17] from patients of all ages between 2001 and 2011. The patients either attended the Dhaka treatment centre of the International Centre for Diarrhoeal Disease Research, Bangladesh (icddr,b) or were referred from public and/or private treatment facilities within Dhaka City and/or outside districts. All isolates were confirmed as *Shigella* spp., by serotyping with commercial *Shigella* antisera (Denka Seiken, Tokyo, Japan) [18]. Of these, 200 *S. sonnei* isolates comprising at least one isolate per month and 20 isolates from each year (except 2008, since no strains were available in our lab stock) were randomly selected for further characterization. The strains were grown in trypticase soy broth containing 0.3% yeast extract and stored at −70°C after addition of 15% glycerol.

### Biotyping

Biotyping was done using standard methods for fermentation of rhamnose and xylose and hydrolysis of ortho-nitrophenyl-β-D-galactopyranoside (ONPG), and biotypes were designated according to methods described elsewhere [19].

### Antimicrobial Susceptibility Test

Antimicrobial susceptibility test was performed by the Kirby-Bauer disc diffusion method on Muller-Hinton agar plates according to the guidelines of the Clinical and Laboratory Standards Institute [20], with commercial antimicrobial discs (Oxoid, Basingstoke, United Kingdom). The antimicrobial discs used in the study were ampicillin (Amp; 10 µg), streptomycin (Str; 10 µg), tetracycline (Te; 30 µg), ciprofloxacin (Cip; 5 µg), nalidixic acid (Na; 30 µg), mecillinam (Mel; 25 µg), sulfamethoxazole-trimethoprim (Sxt; 25 µg), ceftriaxone (Cro, 30 µg), cefotaxime (Ctx; 30 µg), ceftazidime (Caz; 30 µg) and imipenem (Ipm; 10 ug). *E. coli* ATCC 25922 and *Staphylococcus aureus* ATCC 25923 were used as control strains for the susceptibility tests.

### PCR Assay

Detection of *set*1 gene (ShET-1), *sen* gene (ShET-2), *ial* gene, *ipa*H gene, *stx* genes, integrase genes (*int*1, *int*2 and *int*3), class 1 and class 2 cassette regions were performed by polymerase chain reaction (PCR) according to the procedures described previously [1], [21], [22]. The PCR product of representative isolates were sequenced following the procedure described previously [15]. Nucleotide sequences were analysed using BLAST on the website of the National Centre for Biotechnology Information (NCBI). An *Escherichia coli* (ATCC 25922) strain lacking the140 MDa invasive plasmid and showing susceptibility to all antibiotics was used as a negative control in the PCR assays.

### Nucleotide Sequence Accession Numbers

The nucleotide sequences of the gene cassettes in class 1 integron and class 2 integron reported in this paper were submitted to the GenBank using the National Centre for Biotechnology Information (NCBI, Bethesda, MD, USA) under the accession numbers KC964096, KC993867 and KF134652, respectively.

### Plasmid Profile Analysis

Plasmid DNA was prepared according to the alkaline lysis method of Kado and Liu (1981) with some modifications [23], [24]. The molecular mass of the plasmid DNA bands was assessed by comparing with the mobility of known molecular mass plasmids in *E. coli* PDK-9, R1, RP4, Sa and V517 in agarose gels [23].

### Pulsed-Field Gel Electrophoresis

PFGE was performed following the PulseNet protocol using the *Salmonella* Braenderup H9812 strain as molecular weight standard for normalization as recommended previously [25]. Chromosomal DNA was digested with *Xba*I and fragments were separated using a CHEF DR II apparatus (Bio-Rad Laboratories) under the following conditions: switching time from 2.2 to 54.2 s at 6 V cm^−1^ for 20 h at 14°C. The resulting profiles were analysed as described earlier [26].

## Results

### Epidemiologic Studies

**Figure 1:**
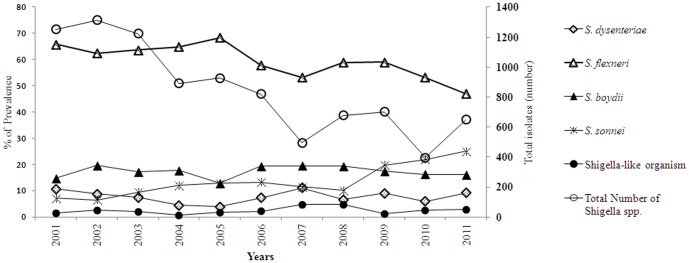
Prevalence of *Shigella* spp. in Bangladesh from 2001 to 2011. The distribution of each *Shigella* species is expressed in percentage and the total isolates of *Shigella* species are expressed in number.

Of 10,827 strains of *Shigella* species isolated from patients of all ages between 2001 and 2011, *S. flexneri* was the dominant species throughout the study period, but the decreasing tendency of predominance was observed starting from 65.7% (824/1252) in 2001 to 47% (306/650) in 2011. At the same time, the prevalence of *S. dysenteriae* came down from 10.7% (135/1252) in 2001 to 9% (60/650) in 2011, whereas *S. sonnei* increased from 7.2% (90/1252) in 2001 to 25% (162/650) in 2011. The frequency of *S. boydii* increased from 14.6% (185/1252) in 2001 to 16.1% (104/650) in 2011 among all age groups of patients in urban areas of Bangladesh (Figure 1). There were a number of strains isolated each year which had characteristics typical of *Shigella* but could not be identified to the species level and were therefore designated as *Shigella*-like organisms (SLOs) which accounted for about 3% of isolates. None of the SLOs agglutinated with any antisera of the established *Shigella* serovars.

### Biotyping

Of 200 strains of *S. sonnei*, 95% (n = 190) were classified as biotype g (ONPG +, rhamnose−, xylose −) and 5% (n = 10) were belonged to biotype a (ONPG +, rhamnose +, xylose −).

### Antibiotic Susceptibility Test

MDR (resistance to ≥ three classes of antimicrobial agents) was detected in 94% (188) of the strains. All the strains were resistant to streptomycin and were susceptible to imipenem, ceftriaxone, cefotaxime, and ceftazidime. Resistance to commonly used antibiotics such as trimethoprim-sulfamethoxazole, nalidixic acid, tetracycline, ciprofloxacin, mecillinam and ampicillin was 89.5%, 86.5%, 84.5%, 17%, 10.5%, and 9.5% respectively. The most prevalent antimicrobial resistance patterns were Str^R^ (100%), Str^R^Sxt^R^ (89.5%), Str^R^Na^R^ (89.5%), Str^R^Te^R^ (84.5%), Str^R^Sxt^R^Te^R^ (83.5%), Str^R^Sxt^R^Na^R^ (79%), Str^R^Sxt^R^Na^R^Te^R^ (74%) and Str^R^Sxt^R^Na^R^Cip^R^ Te^R^ (12%) (Table 1).

**Table 1:** Antibiotic resistance patterns of *S. sonnei* isolated in Bangladesh.

Antimicrobial Resistance Pattern	No (%)
Str	200 (100%)
Str/Sxt	179 (89.5%)
Na/Str	173 (86.5%)
Str/Te	169 (84.5%)
Str/Sxt/Te	167 (83.5%)
Na/Str/Sxt	158 (79.0%)
Na/Str/Sxt/Te	148 (74.0%)
Cip/Str	34 (17.0%)
Cip/Na/Str/Sxt/Te	24 (12.0%)
Mel/Str	21 (10.5%)
Amp/Str	19 (9.5%)
Amp/Str/Sxt/Te	13 (6.5%)
Amp/Na/Str/Sxt/Te	12 (6.0%)

### Distribution of *ipa*H, *sen*, *shet* and Integrons Genes

The *ipa*H gene was detected in all tested strains but none of the strains contained *stx*1 and *set*1 genes, while only 30% (60/200) strains carried both the *ial* and *sen* gene. Of all the strains, 97.5% (195) harbored integrons belonging to either class 1 or 2, or both. Of these strains, two strains carried only class 1 integron (int1), whereas only class 2 integron (int2) was identified in 95% (190) of the strains. None of the strains harbored class 3 integron. Both int1 and int2 integrons were present in three strains (1.5%). Only five strains (2.5%) were lacking both classes of integrons.

### The Resistance Gene Cassettes of Integrons

Using the hep58-hep59 primer pairs, specific for the gene cassettes of int1, one type of gene cassette array (562 bp size) was found in five strains and confirmed as dihydrofolate reductase gene type 5 (*dfr*A5) by DNA sequencing (GenBank accession number KC964096). Two different types of gene cassette arrays were detected using the primer pair hep74-hep51, specific for the gene cassettes of int2. The type-1 1325 bp DNA product of PCR was present in 84% (162/195) of the strains as sequence *dhfr*A1-*sat*2 and 16% (31/195) had 2159 bp band as sequence *dhfr*AI-*sat*1-*aad*A1-*orf*X with the GenBank accession number KC993867 and KF134652, respectively.

### Plasmid Profile Analysis

**Figure 2:**
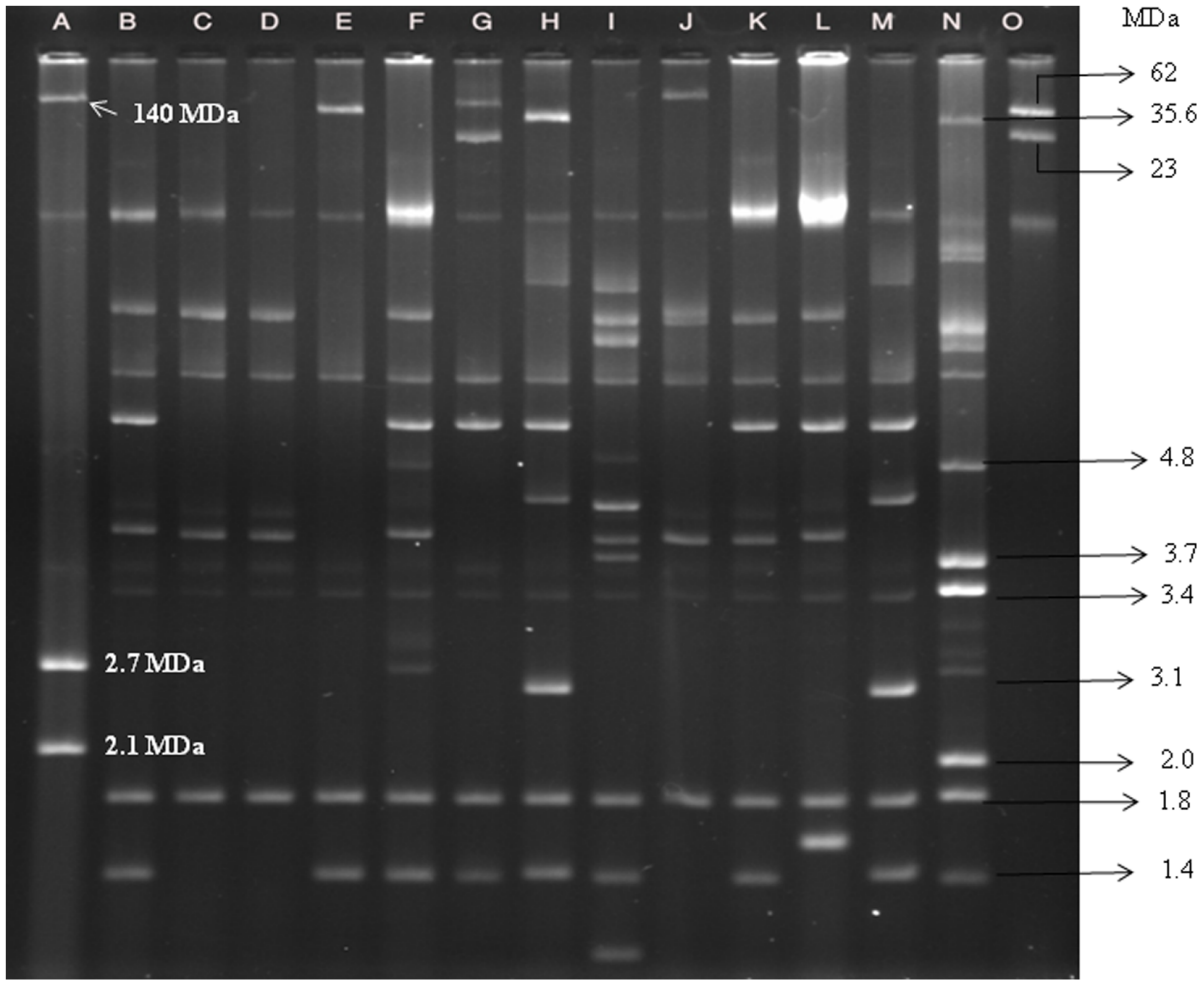
Agarose gel electrophoresis of plasmid DNA showing representative patterns of *S. sonnei*. Lane: A, *E. coli* PDK-9, Lanes: B-M, Representative *S. sonnei* strains, Lane: N, *E. coli* V-517, Lane: O, *E. coli* R-1 and Sa. CHR-indicates the banding position of the chromosome.

Heterogeneous plasmid patterns ranging in size from approximately 120 to 1.0 MDa was found by analyzing plasmid DNA. A number of small plasmids of ∼5, ∼1.8 and ∼1.4 MDa in size were also found to be present universally in more than 92% (184) of the strains and were considered to be the core plasmids of *S. sonnei* (Fig.2). Interestingly, 67% (134) of the strains were found to lack the typical 120 MDa invasive plasmid. A middle-ranged plasmid of approximately 35–90 MDa in size was found in 29% (58) of the strains (Fig. 2).

### PFGE

PFGE analysis of *Xba*I-digested chromosomal DNA of *S. sonnei* strains yielded 15–24 reproducible DNA fragments, ranging in size from approximately ∼20 to 485 kb. These strains yielded four major clusters designated: pulsotype A (n = 88), B (n = 17), C (n = 2) and D (n = 3). The similarity indexes for each cluster were 97.5%, 98.9%, 81.7% and 88.3% respectively. The major pulsotype A was further subdivided into three subtypes (A1–A3) and pulsotype B into two subtypes (B1–B2) (Fig. 3).

**Figure:**
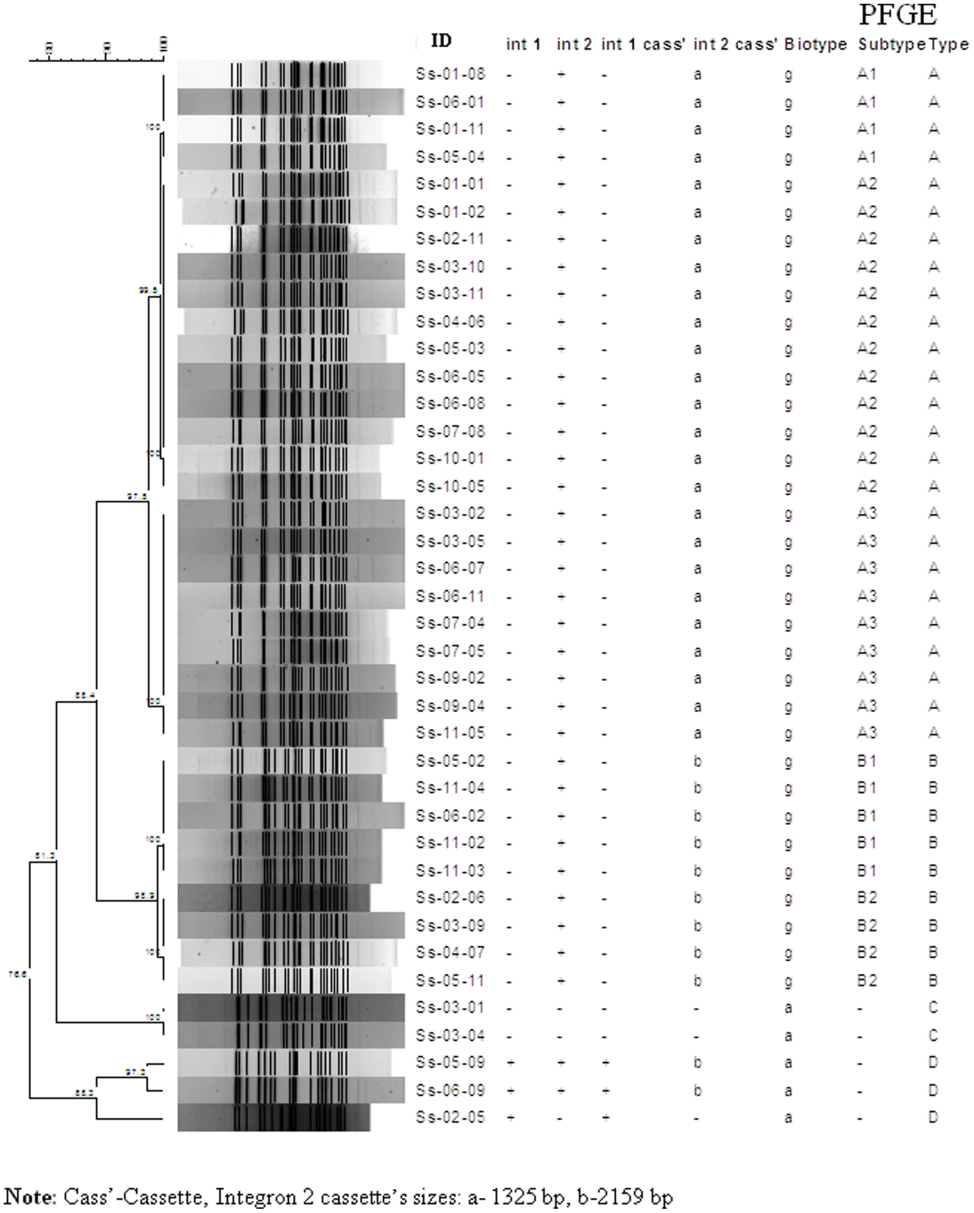
Phylogenetic dendrogram showing the genetic distances among *S. sonnei* using *Xba*I macrorestriction analysis.

### Correlation among Biotype, Integron Cascades and Pulsotypes

All strains belonging to pulsotype A harbored ∼1325 bp int2 and pulsotype B carried ∼2159 bp int2. Strains lacking integrons were grouped into pulsotype C. Pulsotype D contained two strains which harbored both integrons and one strain which harbored only int1. All strains of pulsotypes A and B were biotype g, whereas the rest were biotype a (Table 2).

**Table 2:** Correlation among Biotype, Pulsotypes, Integron Type and Antibiotic Resistance Pattern of *S. sonnei*.

Properties	Class 1 Integron (n = 5)	Class 2 Integron (n = 193)	
Gene cascade size	562 bp (100%, n = 5)	2159 bp (16%, n = 31)	1325 bp (84%, n = 162)
Gene sequence	*dfr*A5 gene	*dhfr*AI*-sat*1*-aad*A1*-orf*X	*dhfr*A1-*sat*2
Biotype	a	g	g
Pulsotype	C	B	A
Resistance pattern	Amp, Str, Sxt	Str, Sxt, Te	Mixed patterns

## Discussion

This study was done for a baseline assessment of the epidemiological features of *Shigella* species, with special emphasis on characterization of the recent endemic *S. sonnei* in Bangladesh. A changing trend in the distribution of *Shigella* species was found in this study. The rising trend of shigellosis due to *S. sonnei* (7.2% to 25%) and *S. boydii* (14.8% in 23to 16.1%), and decreasing trend due to *S. flexneri* (65.8% to 47%) and *S. dysenteriae* (10.8% to 9.2%) was observed in urban areas of Bangladesh from 2001 to 2011. In the past, from 1999 to 2003 about 8% of shigellosis cases per year was caused by *S. sonnei* [21]. Since then the increasing trend was seen from ∼12% in 2004 to ∼25% in 2011 in urban area of Bangladesh. The increasing trend of *S. sonnei* from 35% (50/144) in 2010 to 41% (63/154) in 2012 was also observed in another study conducted in rural areas of Bangladesh (Talukder *et al*., unpublished data). Similar trend was noticed in many developed and industrialized countries [27] including India [28] and Pakistan [29]. The reasons for this shifting trend have been suggested to be an improvement of overall nutritional status [8], [30], socioeconomic status [31], sanitation condition [32], and a reduction of the chances of cross immunity imparted by *Plesiomonas shigelloides* [33].

In the present study, we found that biotype g (rhamnose non-farmenter) was the predominant (95%) biotype followed by biotype a (rhamnose farmenter). It is interesting to note that all strains were found to be rhamnose farmenter in an earlier study [21]. Recent emergence of biotype g strains has also been reported from the developing and industrialized countries including Ireland [4], Italy [34], Australia [35], Malaysia [36] and South Korea [37]. Therefore, biotype g appears to have emerged and spread globally in recent decades contributing to the prevalence of this biotype throughout the world [34], [38]–[40].

Antimicrobial resistance has emerged as a serious global public health concern. In low and middle income countries including Bangladesh, the declining susceptibility to commonly used antibiotics and emergence of MDR bacteria have been linked to the indiscriminate or inappropriate use of antibiotics. The other contributing factors include bacterial evolution, climate changes, cheap and ready availability of antibiotics, lack of medical practitioner with proper training, poor-quality of available drugs and unhygienic sanitary conditions [32], [41], [42]. The majority of the strains in this study were resistant to commonly used antibiotics including Na, Str, Sxt and Te. We detected that resistance of *S. sonnei* to Cip was about 10% in 2007 and increased dramatically by sevenfold (70%) in 2011. In Bangladesh, resistance of *S. dysenteriae* 1 to Cip was first detected as early as in 2003 but in case of *S. flexneri* and *S. boydii* it was detected in 2007 and 2008, respectively [8], [43]. In the present study, all the tested strains were susceptible to 3^rd^ generation cephalosporins (Cro, Ctx and Caz) and Ipm probably due to their very infrequent use as alternative therapeutic regimens in this geographic area. Notably, Ipm resistance was found in other enteric pathogens except *Shigella* spp. [44].

Plasmid profile analysis is a well documented and important tool for epidemiological studies of enteric pathogens [3]. It has been reported earlier that *S. sonnei* strains possessed multiple numbers of plasmids with a heterogeneous combination but three plasmids of ∼5, ∼1.8 and ∼1.4 MDa (core plasmids) were commonly present (90%) in most *S. sonnei* [21]. Of 200 strains analyzed in this study, 92% contained these core plasmids supporting our previous report [21] and 33% of the strains contained both the *sen* and *ial* gene and the typical 120-MDa invasive plasmid. The possible explanations for the absence of the 120 MDa plasmid might be a loss of the plasmid due to (i) long-term storage at −70°C and (ii) repeated subculturing of the strains [45]. Self-transmissible middle-range plasmids (∼30–90 MDa) in *Shigella* spp. are associated with antibiotic resistance [46]. In this study only 17% of the MDR strains had middle-range plasmids. Most *Shigella* strains harbored one or two classes of integrons. The presence of integrons in *Shigella* species varied in numbers in diverse geographical regions [1], [4], [26], [47], [48]. The apparent correlation between multidrug resistance and presence of integron in the strains was not due to the presence of integrons on conjugative plasmids. Integrons found in most strains were chromosomal, and thus, it can be concluded that antibiotic resistance was not only due to the presence of middle-range plasmids but also for the presence of chromosome mediated genes [1].

The low prevalence (2.5%) of int1 found in this study was also detected in other countries [16], [48]. In this study we found that only *dfr*A5 gene in int1 cassette conferred trimethoprim resistance, an observation rarely reported in *S. sonnei* [49]. Several investigators reported the presence of this gene in *S. flexneri* and other members of *Enterobacteriaceae* [48], [50]. The presence of int2 was significantly higher in MDR strains (p <0.001). Although other mechanisms are possible, resistance to streptomycin, spectinomycin and trimethoprim seems to be attributable to expression of genes contained in int2 of the members of Tn7 family. Generally, int2 carries the open reading frames *dhfr*I, *sat*1 and *aad*A. However, due to the presence of a defective integrase, some different structures have been very infrequently found in endemic *S. sonnei* strains [4], [48]. This study showed a higher prevalence (16%) of int2 of ∼2159 bp in size harboring *dhfr*I, *sat*1 and *aad*A gene compared to previous studies. Strains harboring ∼2159 bp integron showed uniform resistance to Str, Sxt and Te, whereas no specific association with antibiotic resistance pattern was found in strains containing ∼1325 bp class 2 integron. The same array of gene cassettes of int2 was found in other members of *Enterobacteriaceae* [38], [51]. The variation of int2 cassette content among the MDR *S. sonnei* strains from different geographical locations might be due to the independent acquisitions of the integron over time and further diversification by clonal expansion and subsequent global spreading [40].

To date, a number of different genotyping methods including ribotyping, inter-IS1 spacer typing (IST) method, enterobacterial repetitive intergenic consensus sequence based PCR, multilocus variable-number tandem-repeat analysis (MLVA) and PFGE have been used for the epidemiological investigation and phylogenetic study of *S*. *sonnei* [26], [52], [53]. Although MLVA has good discriminatory power for distinguishing epidemiologic relationship, it is not a universal method and not 100% reproducible, and has no properly validated protocol for use in surveillance networks [54]. On the other hand, PFGE is the gold standard, has well-established global surveillance network and has been used successfully for the investigations of outbreaks as well as epidemiological studies of *Shigella* species [21]. PFGE analysis of these strains showed that *S. sonnei* biotype a strains were genetically more diverse than biotype g strains, and revealed that strains having different integron patterns belonged to different clusters. This finding is congruent with a previous study [38].

Overall, our findings suggest a decrease in prevalence of *S. flexneri* and at the same time a drastic increase in *S. sonnei* to become the second predominant species by replacing *S. boydii* and *S. dysenteriae* in Bangladesh. *S. sonnei* biotype g carrying int2 was prevalent. The high prevalence of integrons in *Shigella* may have clinical significance, as multiple gene cassettes could be integrated into integrons leading to multidrug resistance, even to broad-spectrum antibiotics such as cephalosporins and quinolones among *Shigella* species. Development of a suitable vaccine against *S. sonnei* should be a priority in order to reduce the disease burden due to *Shigella* infection in Bangladesh, as all *S. sonnei* share a common O antigen that has proven to be a potential vaccine candidate.
